# New Drugs. Appliances, and Things Medical

**Published:** 1892-04-09

**Authors:** 


### NEW DRUGS, APPLIANCES, AND THINGS MEDICAL.

[All preparations' appliances, novelties, etc., of which a notice is desired, should be sent for The Editor, to care of The Manager, 140, Strand, London, W.C.]

### SEDOX ABSORBENT DRESSINGS.

We have received from the Sedox Manufacturing Company, 12, Oat Lane, E C., the following samples of dressings prepared from this vegetable fibre. The plain absorbent, corrosive sublimate, sal alembroth, salicylic acid, boric acid, also accouchement sheet and a respirator. It is claimed for this material, first, that it absorbs 15 times its weight of fluid. Secondly, that because of the ease of its manufacture it is cheaper than cotton wool, &c., in the proportion of 1 to 1·5. Thirdly, in consequence of the length of the fibre there is little or no adhesion to wounds. Fourthly, that it is extremely soft and elastic, and so is pre-eminently comfortable to the patient. Now we always test statements like these as far as we can practically, so three portions of the simple absorbent were taken, weighed dry, and then after well soaking in water, weighed wet.

| | Dry weight | | After immers'on | |
|---|---|---|---|---|
| (*a*) | 147 grms. | ... | 2131·5 grms. | =14·5 |
| (*b*) | 138 ,, | ... | 2107 ,, | =15·25 |
| (*c*) | 151 ,, | ... | 2254·43 ,, | =14·93 |

This then well bears out the first claim. The second claim is obvious to all who purchase this class of goods. The third we find also to be well borne out; the dressing does not stick to wounds. Fourthly, the patients who were dressed with this material (in several forms) expressed themselves as well pleased with its comfort and ease. The various medicated dressings were all tested for the respective drugs which were found to be present. The accouchement sheet has a waterproof side, so that no soaking through occurs, and as it is burnt after use, it seems to us to be an extremely useful and highly sanitary necessary to the lying-in room. The respirator is light and, as far as respirators can be called so, an elegant appliance. From its cheapness it can also be burnt on becoming soiled, a great desideratum in phthisical and other infectious or offensive pulmonary complaints. We believe that sanitary towels and other useful appliances are also manufactured by the company. In conclusion we consider that the company has made out its claims, and that it has added another most useful material to the list of surgeons' dressings.

